# Bacterial Cheaters Evade Punishment by Cyanide

**DOI:** 10.1016/j.isci.2019.07.015

**Published:** 2019-07-16

**Authors:** Parker Smith, Jamison Cozart, Bryan K. Lynn, Erin Alberts, Emanuela Frangipani, Martin Schuster

**Affiliations:** 1 Department of Microbiology, Oregon State University, Corvallis, OR 97331, USA; 2 Department of Integrative Biology, Oregon State University, Corvallis, OR 97331, USA; 3 Department of Biomolecular Sciences, University of Urbino “Carlo Bo”, 61029 Urbino (PU), Italy

**Keywords:** Biological Sciences, Genetics, Microbiology, Evolutionary Biology

## Abstract

In all domains of life, mechanisms exist that protect cooperating groups from exploitation by cheaters. Recent observations with the bacterium *Pseudomonas aeruginosa* have suggested a paradigmatic cheater control mechanism in which cooperator cells punish or “police” cheater cells by cyanide poisoning. These cheater cells are deficient in a pleiotropic quorum-sensing regulator that controls the production of cooperative secretions including cyanide, and presumably also cyanide resistance. In this study, we directly tested and refuted the cyanide policing model. Contrary to the hypothesis, cheater fitness was unaffected by the presence of cyanide. Cheater mutants grew equally well in co-cultures with either cyanide-proficient or cyanide-deficient cooperators, and they were as resistant to exogenous cyanide as wild-type cells. We show that these behaviors are the result of quorum-sensing-independent and cyanide-responsive resistance gene regulation. Our results highlight the role of genetic architecture in the evolution of cooperative behavior.

## Highlights

• The cyanide policing hypothesis in *Pseudomonas aeruginosa* is refuted
• Cyanide does not restrict the growth of non-cooperating cheater mutants
• Cyanide production and resistance are regulated by independent pathways
• Genetic architecture influences the maintenance of cooperation

## Graphical Abstract

**Figure:**
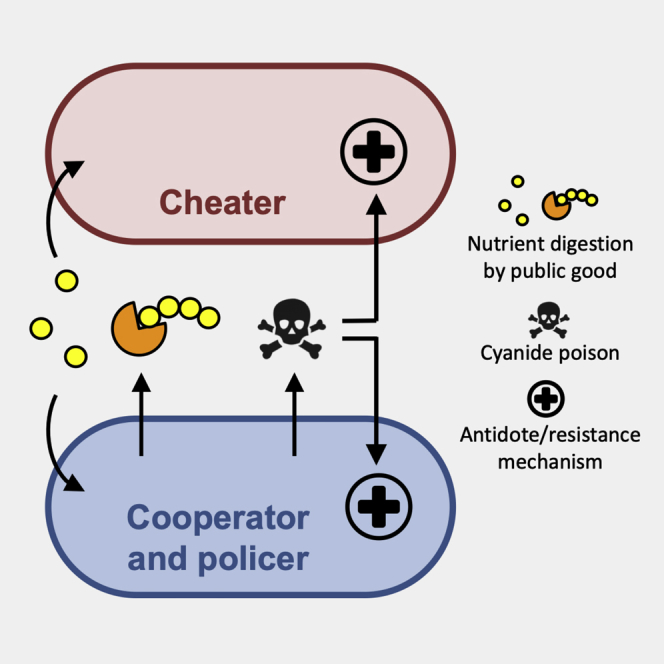

## Introduction

Cooperative behavior is common in all domains of life, although it is intrinsically vulnerable to exploitation by non-cooperating cheats (Hamilton, 1964, Hardin, 1968). Thus, there has been great interest in identifying mechanisms that maintain cooperation. A range of different cheater control mechanisms have been described, including limited dispersal, kin discrimination, enforcement, and pleiotropy (Foster et al., 2004, Foster et al., 2007, Ratnieks et al., 2006, Schuster et al., 2013, Travisano and Velicer, 2004). Policing is a type of enforcement in which cooperators punish cheaters (El Mouden et al., 2010, Frank, 1995). Worker policing in the honeybee is the prototypical example. Here, workers suppress the reproduction of other, selfish workers by eating their eggs (Ratnieks, 1988).

The objective of this study is to investigate policing in bacteria. Bacteria exhibit a wide variety of cooperative activities such as biofilm formation, nutrient acquisition, and quorum sensing (QS) (West et al., 2006). Bacterial cooperation generally involves the secretion of shared products referred to as “public goods” (West et al., 2007). The model bacterium and opportunistic pathogen *Pseudomonas aeruginosa* secretes many public goods that are under the control of a cell-cell signaling circuitry termed quorum sensing (Lee and Zhang, 2015, Schuster et al., 2013, Williams and Camara, 2009). Two hierarchically arranged acyl-homoserine lactone QS systems, LasR-LasI and subordinate RhlR-RhlI, control the expression of about 300 genes (Schuster et al., 2003, Wagner et al., 2003). LasI and RhlI are signal synthases that produce 3-oxo-dodecanoyl-homoserine lactone and butanoyl-homoserine lactone, respectively (Pearson et al., 1994, Pearson et al., 1995). LasR and RhlR are the cognate signal receptors that activate transcription of target genes (Ochsner and Reiser, 1995, Passador et al., 1993). The public goods controlled by the two systems include proteases that digest extracellular protein as nutrient source and hydrogen cyanide that poisons other cells (Brint and Ohman, 1995, Pearson et al., 1997, Schuster et al., 2003). Factors that contribute to cyanide resistance are also affected by QS (Frangipani et al., 2014, Schuster et al., 2003).

QS-controlled proteolysis in *P. aeruginosa* is a well-studied example of a bacterial trait susceptible to cheating (Asfahl and Schuster, 2017, Schuster et al., 2013). Cheaters evolve in the form of signal-blind *lasR* mutants that reap the benefits of proteolysis without contributing to the costs (Dandekar et al., 2012, Diggle et al., 2007, Kohler et al., 2009, Sandoz et al., 2007). During experimental evolution of *P. aeruginosa* in a protein medium, *lasR* mutants enrich quickly within the first 100 generations, but then reach an apparent equilibrium with the cooperating wild-type (Asfahl et al., 2015, Dandekar et al., 2012, Sandoz et al., 2007). The fact that cheating does not lead to a population collapse has primed research into possible cheater control mechanisms. One such mechanism is the acquisition of non-social mutations in cooperators that increase the cellular uptake of digested peptides (Asfahl et al., 2015). A second proposed mechanism is the policing of *lasR*-deficient cheaters by wild-type cooperators (Wang et al., 2015). According to this model, *lasR*-deficient mutants are susceptible to the cyanide produced by wild-type cells, because LasR pleiotropically links protease production to cyanide production and resistance (Wang et al., 2015). Although this model is intriguing and has received considerable attention (e.g., Asfahl and Schuster, 2017, Defoirdt, 2018, Ozkaya et al., 2017, Wechsler et al., 2019, Whiteley et al., 2017, Yan et al., 2018), it cannot explain the large initial fitness advantage of *lasR* mutants, and it is primarily based on indirect evidence. Conclusions are mainly drawn from genetically undefined evolution experiments, without information about cyanide concentrations and the effects of exogenous cyanide. Moreover, cyanide production requires specific conditions and the regulation of resistance is complex.

Cyanide is maximally produced at high cell density and under low oxygen conditions (Pessi and Haas, 2000). The *hcnABC* operon encoding HCN synthase is activated synergistically by LasR, RhlR, and the anaerobic regulator ANR (Pessi and Haas, 2000). Cyanide resistance is mediated by different mechanisms. One involves intracellular inactivation of cyanide by the enzyme rhodanese (Cipollone et al., 2007). Another involves the overlapping action of the cyanide-insensitive cytochrome *bd* quinol oxidase CIO, encoded by *cioAB* (Cunningham et al., 1997, Zlosnik et al., 2006), and the products of a six-gene cluster, PA4129-PA4134 (Frangipani et al., 2014). Genes in this cluster encode the alternative, cyanide-insensitive subunit CcoN4 of a *ccb*_*3*_ cytochrome *c* oxidase, other proteins involved in electron transfer, and proteins of unknown function (Frangipani et al., 2014, Hirai et al., 2016). CIO and oxidase isoforms containing CcoN4 differ in their oxygen affinity and level of cyanide resistance (Arai et al., 2014, Hirai et al., 2016, Zlosnik et al., 2006).

The expression of *cioAB* and of genes in the PA4129-34 cluster is triggered by endogenous (self-produced) and exogenous (added) cyanide to varying degrees (Cooper et al., 2003, Frangipani et al., 2014, Hirai et al., 2016). The expression of PA4129-34 is also induced by LasR, albeit likely indirectly (Gilbert et al., 2009, Schuster et al., 2003, Wurtzel et al., 2012), and possibly as a result of LasR-dependent activation of cyanide production. Thus, available data on gene regulation suggest that cyanide might trigger resistance even in *lasR*-deficient cheaters.

In this study, we directly tested the predictions made by the cyanide policing model. We co-cultured *lasR* cheaters with cyanide-producing and non-producing cooperators under defined conditions, quantified cyanide levels, examined the inhibitory effect of exogenous cyanide, and investigated the underlying regulation of resistance genes PA4129–34 and *cioAB*. Taken together, we find that *lasR* cheaters evade policing by cooperating wild-type cells. The cheaters are resistant to cyanide because resistance gene activation is independent of QS and is in large part triggered by cyanide directly. We discuss broader implications for the evolution of cooperation and associated control mechanisms.

## Results

### Cooperators Do Not Police Cheaters in Defined Co-culture

As the first step, we tested the hypothesis that cyanide produced by the wild-type cooperator reduces the fitness of the *lasR* cheater in co-culture. We used an established growth medium with casein as the sole, QS-dependent carbon and energy source (Asfahl et al., 2015, Sandoz et al., 2007, Wilder et al., 2011). We varied cyanide concentrations in cultures in two ways. First, we used either the cyanide-producing wild-type or a defined *hcnB* deletion mutant that does not produce any cyanide (Frangipani et al., 2014). Second, we grew cultures under either high or low aeration by varying agitation speeds and tube closures. High-aeration conditions are analogous to all previous work, including the initial policing study (Wang et al., 2015). Low-aeration conditions are predicted to increase cyanide concentrations by enhancing its production and preventing the escape of cyanide gas (Frangipani et al., 2014).

**Figure 1:**
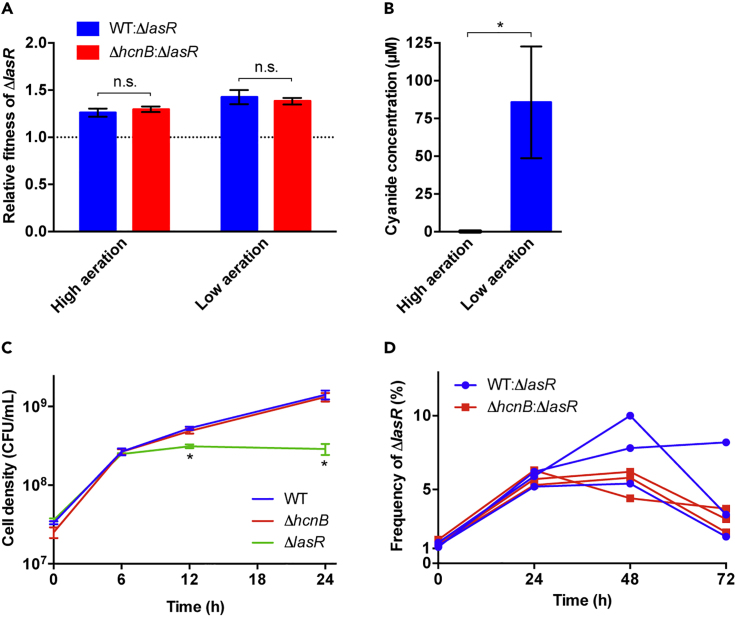
Effect of Endogenous Cyanide Production In Co-cultures Cultures of *P. aeruginosa* strains were grown in casein minimal medium. Growth and cyanide levels were measured as indicated. (A) Relative fitness of the Δ*lasR* mutant in co-culture with the wild-type (WT, blue bar) or the Δ*hcnB* mutant (red bar) in high- and low-aeration conditions. Co-cultures were initiated with the Δ*lasR* mutant at approximately 1% frequency and grown for 24 h. Initial and final cell counts of each strain were determined, and relative fitness was calculated as the ratio of average growth rates. A relative fitness greater than 1 indicates enrichment of the Δ*lasR* mutant. Relative fitness was significantly above 1 in all co-cultures (one-sample t test, p < 0.01), but was not significantly different between the WT:Δ*lasR* and Δ*hcnB*:Δ*lasR* co-cultures at low and high aeration (n.s., two-way ANOVA with post-hoc test, p = 0.71 and p = 0.81, respectively). (B) Cyanide production of the WT:Δ*lasR* co-culture in the high- and low-aeration conditions as described in (A). Cyanide concentrations were measured after 24 h of growth, using a cyanide-negative Δ*hcnB* mutant culture as background control; hence, cyanide production was not determined for the Δ*hcnB*:Δ*lasR* co-cultures. Cyanide concentrations were significantly different between co-cultures (asterisk, two-sample t test, p = 0.016). (C) Cell densities (colony-forming units [CFU]/mL) of the WT (blue line), the Δ*hcnB* mutant (red line), and the Δ*lasR* mutant (green line) grown for 24 h in individual cultures with low aeration. Only the Δ*lasR*-mutant densities were found to be significantly different from the other two strain densities at 12 and 24 h (asterisks, two-way ANOVA with post-hoc test, p < 0.02). (D) The frequency of Δ*lasR-*mutant cells in co-culture with the WT (blue line with circles) or the Δ*hcnB* mutant (red line with squares) over a 72-h period. Co-cultures were grown at low aeration and sub-cultured into fresh medium every 24 h. The Δ*lasR* mutant was at approximately 1% initial frequency. Each value is the measurement of a single replicate. Three replicates of each co-culture are shown. In (A–C) data points are the means of three independent biological replicates, with error bars indicating standard deviation. Complete statistical data are available in Tables S1–S4.

We initiated wild-type*:lasR* and *hcnB:lasR* co-cultures at a ratio of 99:1. The low initial *lasR* mutant frequency simulates the emergence of a cheater in a cooperating population and affords high relative fitness to the cheater, which in turn increases the ability to discern differences between experimental conditions. We restricted the duration of co-culture growth to a single 24-h period, because prolonged culturing is known to select for additional mutations that may confound interpretation of the data (Asfahl et al., 2015). We found that *lasR* mutant fitness is greater than one in all co-cultures, confirming the cheater phenotype (Figure 1A). Importantly, the *lasR* cheater had the same fitness in wild-type and *hcnB* co-cultures, regardless of aeration. However, aeration had a large effect on cyanide production, consistent with previous reports (Frangipani et al., 2014, Pessi and Haas, 2000). Cyanide accumulated to high levels under low aeration, but was undetectable under the high-aeration conditions previously reported to promote policing (Figure 1B).

It is conceivable that the detrimental effect of cyanide on the *lasR* mutant is masked in part by a concomitant reduction in wild-type growth rate due to the cost of cyanide production. To test this possibility, we grew individual cultures of the *hcnB* mutant and the wild-type parent in casein medium under low aeration. We found that their growth was indistinguishable, suggesting that there is no measurable cost to cyanide production (Figure 1C). This outcome is consistent with the low cost of individual cooperative traits reported in another study (Mitri and Foster, 2016). For comparison, we also grew the *lasR* mutant in this medium, demonstrating its reduced fitness under conditions that favor QS. Of note, the initial growth of the *lasR* mutant at low density is consistently observed in this medium and is presumably due to the presence of breakdown products in the commercial casein stock that can be utilized without QS (Asfahl et al., 2015, Sandoz et al., 2007, Wilder et al., 2011).

It is possible that a single, 24-h growth cycle is insufficient to reveal a very small effect of cyanide policing on cheater fitness. To examine this notion, we grew the wild-type*:lasR* and *hcnB:lasR* co-cultures for three consecutive 24-h cycles, with sub-culturing every 24 h (Figure 1D). Even here, we found no evidence of policing. However, beyond the first 24 h, individual replicates appeared to have different evolutionary trajectories, consistent with our previous finding that there is strong selection for additional, beneficial mutations in this environment (Asfahl et al., 2015). These adaptations can also explain why the proportion of *lasR* mutants stagnates or even decreases in most replicates after the first 24 h. By chance alone, mutations are much more likely to be sampled by the initially abundant population, providing a selective advantage to the cooperator over the cheater (Waite and Shou, 2012).

### Exogenous Cyanide Has No Effect on Cheater Growth

**Figure 2:**
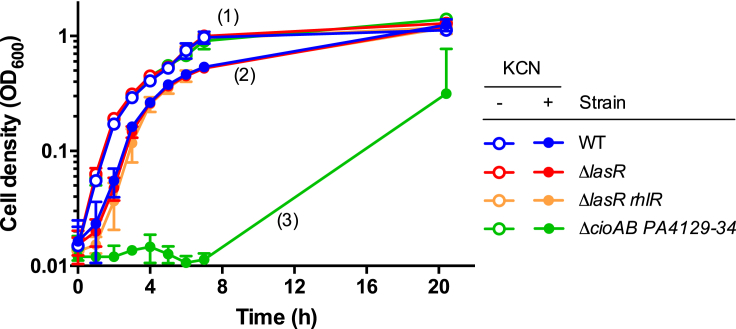
Effect of Exogenous Cyanide on Individual Cultures The *P. aeruginosa* wild-type (WT), Δ*lasR*, Δ*lasR rhlR*, and cyanide-sensitive Δ*cioAB* PA4129-34 strains were grown in individual LB cultures under low aeration, with and without exogenous cyanide as indicated. Cyanide was added at 100 μM final concentration upon inoculation, and growth was measured as optical density at 600 nm (OD_600_) over time. Significant differences between strain densities at each time point were assessed by two-way ANOVA with post-hoc test, allowing the distinction of three groups as indicated: (1) all four strains without exogenous cyanide; (2) WT, Δ*lasR*, and Δ*lasR rhlR* with exogenous cyanide; and (3) Δ*cioAB* PA4129-34 with exogenous cyanide by itself. Cell densities differed significantly between strains from different groups, but not between strains within the same group (see Tables S1 and S5 for individual p values). Data points are the means of three independent biological replicates, with error bars indicating standard deviation. In one case, the bottom arm of an error bar is missing, because it has a negative value, and a negative value cannot be plotted on a log scale.

To directly demonstrate the effects of exogenous cyanide on *lasR* cheater growth, we cultured *P. aeruginosa* strains individually in a complex medium (lysogeny broth [LB]) used in our previous study to assess cyanide sensitivity (Frangipani et al., 2014). This medium permits equal growth of wild-type and QS mutant strains, because it does not require QS-controlled proteolysis (Schuster et al., 2003). In addition to the wild-type and the *lasR* mutant, we included a *lasR rhlR* double mutant in the analysis, to exclude the possibility that RhlR might activate cyanide production or resistance even in the absence of LasR. As a negative control, we included the *cioAB* PA4129-34 double mutant, which is sensitive to cyanide under the growth conditions employed, whereas the respective single mutants are resistant (Frangipani et al., 2014). We added cyanide at 100 μM final concentration, which is similar to that measured in casein medium under low aeration (Figure 1B). We found that the wild-type and the two QS mutants (*lasR* and *lasR rhlR*) grow more slowly in the presence of cyanide, but we also found that the three strains grow equally well (Figure 2). For comparison, the cyanide-sensitive double mutant was severely impaired under these growth conditions, confirming that cyanide levels are sufficiently high. Taken together, these data provide additional evidence that cyanide has no effect on *lasR* mutant growth.

### Resistance Gene Expression Is QS Independent

To investigate the underlying regulatory mechanism of cyanide resistance, we quantified the expression of resistance genes PA4129-34 and *cioAB*. The PA4129-34 gene cluster is predicted to be organized into three transcript units (PA4130-29, PA4131-32, and PA4133-34), with promoters upstream of PA4130, PA4131, and PA4133, respectively (Frangipani et al., 2014). We reasoned that the expression of resistance genes would be triggered by cyanide even without a functional QS system, affording resistance to the *lasR*-mutant cheater. To examine this possibility, we compared gene expression between the wild-type, the *lasR* mutant, and the *hcnB* mutant in low-aeration LB cultures, either with or without exogenous cyanide. Cultures were grown to high density in early stationary phase such that the contribution of QS to the induction of resistance genes could be assessed. We added cyanide at a final concentration of 25 μM. This lower concentration was chosen according to our previous study to minimize effects on growth while retaining induction of cyanide resistance genes (Frangipani et al., 2014). We used β-galactosidase reporters fused to three promoters, PA4130’-’*lacZ*, PA4133’-‘*lacZ*, and *cioAB*’-‘*lacZ*. We did not include the PA4131 promoter here because previous work indicated that the PA4131 start codon is wrongly annotated (Frangipani et al., 2014). We related cyanide resistance gene expression to endogenous cyanide synthesis by also monitoring *hcnABC* expression using an *hcnA*’-‘*lacZ* fusion.

**Figure 3:**
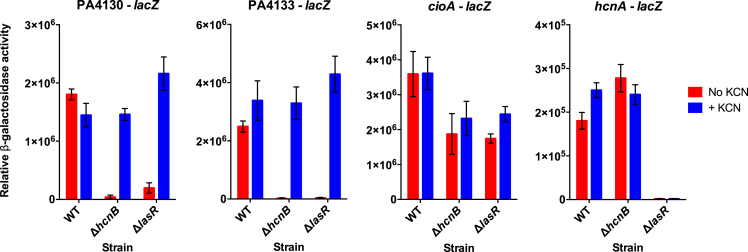
Expression of Cyanide Resistance and Production Genes β-Galactosidase activities of PA4130’-‘*lacZ*, PA4133’-‘*lacZ*, *cioA’-‘lacZ*, and *hcnA*’-‘*lacZ* reporter fusions were measured in the *P. aeruginosa* wild-type (WT), the Δ*hcnB* mutant, and the Δ*lasR* mutant, using a luminescence assay. Cultures were grown in LB medium under low aeration until early stationary phase (OD_600_ of 1.0–1.5), with or without 25 μM potassium cyanide (blue and red bars, respectively). Relative β-galactosidase activities represent luminescence values normalized to OD_600_. Data points are the means of three independent biological replicates, with error bars indicating standard deviation. See Tables S1 and S6 for complete statistics and pairwise comparisons.

We found that cyanide has a dramatic, QS-independent effect on the expression of PA4130’-‘*lacZ* and PA4133’-‘*lacZ* (Figure 3). Exogenous cyanide highly induced expression in cyanide-deficient *hcnB* and *lasR* mutants, whereas endogenous cyanide induced expression to similar levels in the wild-type. Without exogenous cyanide, PA4130’-‘*lacZ* and PA4133’-‘*lacZ* expression was lowest in the QS-proficient *hcnB* mutant, indicating that QS itself is insufficient to induce these resistance genes. In contrast, endogenous and exogenous cyanide had a much smaller (maximally 2-fold) effect on the expression of *cioA*’-‘*lacZ*. Expression levels were comparatively high in the mutant strains, consistent with the largely QS and cyanide-independent induction of *cioAB* in stationary phase observed previously (Cooper et al., 2003, Schuster et al., 2003). Exogenous cyanide caused a slight induction of *cioA’-‘lacZ* in the *lasR* mutant. Endogenous cyanide production likely contributed to the full induction seen in the wild-type, given the nearly identical expression levels of the non-producing *lasR* and *hcnB* mutants. Finally, QS had a strong effect on cyanide production irrespective of cyanide addition, inducing *hcnA*’-‘*lacZ* expression about 100-fold in the wild-type and the *hcnB* mutant compared with the *lasR* mutant, consistent with previous reports (Pessi and Haas, 2000). Taken together, our data indicate that cyanide and other QS-independent cues are sufficient to trigger the expression of resistance genes, explaining the ability of QS-deficient cheaters to evade policing by cyanide-producing wild-type cells.

## Discussion

Microbes exhibit a wide range of cooperative behaviors. They provide excellent model systems to experimentally investigate the mechanisms that restrain cheating and stabilize cooperation (West et al., 2007). Many different mechanisms have been described, including spatial structure, population dispersal, metabolically prudent regulation, partial privatization of public goods, and environmental adaptation (Asfahl and Schuster, 2017). Another intriguing mechanism that has been proposed in the opportunistic pathogen *P. aeruginosa* is the policing of QS-deficient cheaters by cyanide-producing cooperators (Wang et al., 2015). Cheating in *P. aeruginosa* has been shown in experimental infection, is clinically relevant, and may be useful as a novel anti-infective strategy (Allen et al., 2014, Brown et al., 2009, Kohler et al., 2009, Rumbaugh et al., 2009).

In this study, we directly examined and refuted the central assertions of the cyanide policing model by complementary approaches. We showed that cyanide production by wild-type cooperators has no effect on the growth of *lasR*-mutant cheaters in co-cultures (Figure 1), and we showed that *lasR* mutants are as resistant to exogenous cyanide as the wild-type in individual cultures (Figure 2). Of course, the accumulation of endogenous cyanide during growth is different from the provisioning of cyanide at the beginning of growth. Nevertheless, our cyanide addition experiment allows us to conclude that even sustained exposure of growing cultures to high levels of cyanide has no effect on cheater fitness.

**Figure 4:**
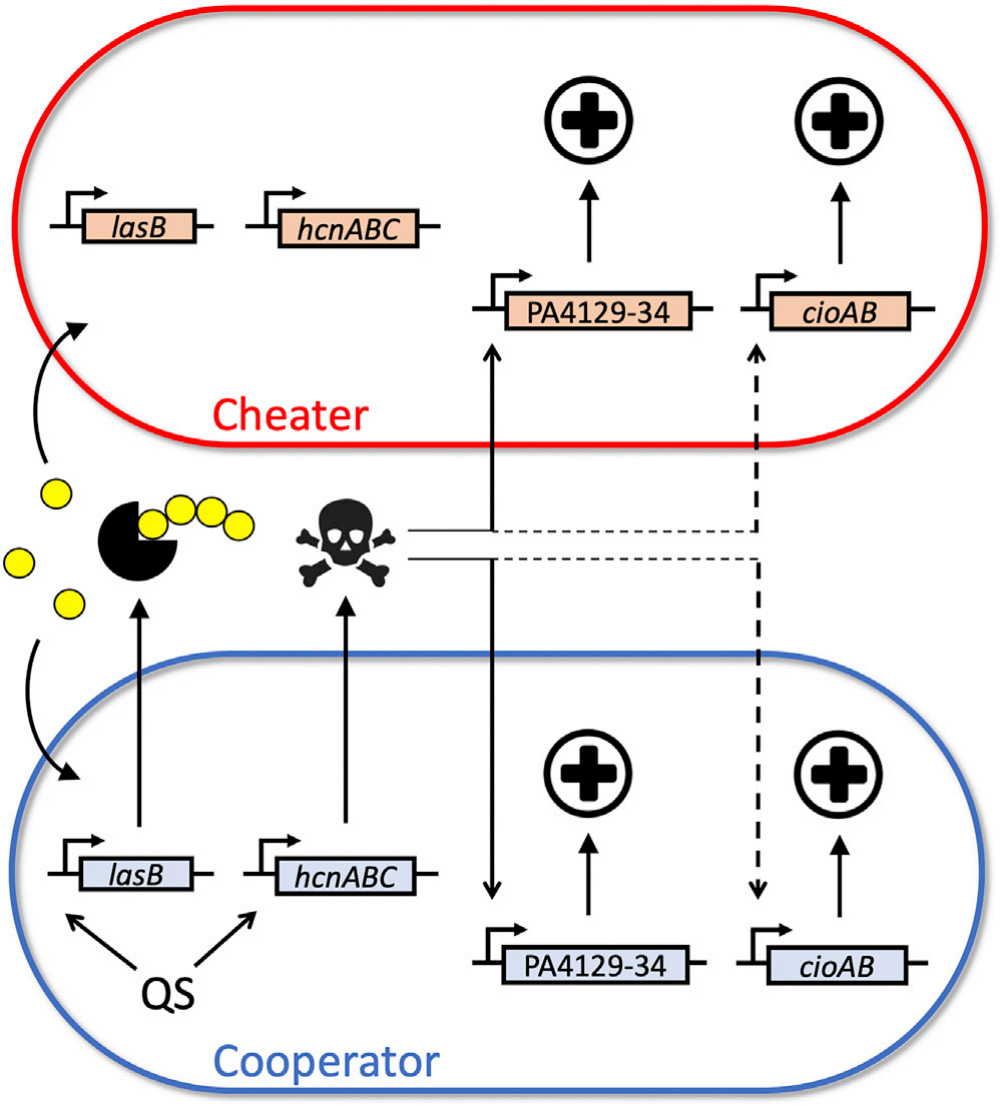
Model of Cyanide Production and Resistance in Cooperator and Cheater Cells QS directly activates, via LasR and RhlR, the expression of *lasB* and *hcnABC* in the cooperator. The secreted protease LasB elastase (Pac man), together with other proteases, digests peptides (yellow chain) that serve as nutrient source to both the cooperator and the cheater. The *hcnABC* genes lead to production of diffusible cyanide (skull) by HCN synthase. Genes PA4129-34, shown here for simplicity as single contiguous piece, and *cioAB* encode cyanide resistance determinants (cross). PA4129-34 and to a lesser extent *cioAB* (solid and dashed arrows, respectively) are directly activated by cyanide but not by QS. Consequently, cyanide resistance is achieved in both QS-proficient cooperator and QS-deficient cheater cells.

We then demonstrated that the high *lasR* cheater fitness can be explained by the specific regulation of resistance genes (Figure 3). Two complementary loci involved in resistance, *cioAB* and PA4129-PA4134, are induced by endogenous and exogenous cyanide without the direct contribution of QS (see model in Figure 4). The *cioAB* promoter was less responsive to cyanide than PA4129-PA4134, probably as a consequence of lower cyanide sensitivity overall and a masking effect from strong cyanide and QS-independent induction of *cioAB* in stationary phase (Cooper et al., 2003). Our data suggest that the previously reported QS dependence of PA4129-34 (Schuster et al., 2003) is largely an indirect effect of QS-controlled cyanide expression: LasR and RhlR induce *hcnABC* expression, resulting in the production of cyanide, which in turn induces PA4129-34. The same is likely true for the modest *lasR*-dependent induction we observed for *cioAB*. In contrast to PA4129-34, *cioAB* was not identified as QS dependent in our previous microarray study (Schuster et al., 2003). However, reinterrogation of these data indeed shows a small 1.5- to 2-fold difference in transcript levels that was below the cutoff chosen (Schuster et al., 2003). The conclusion that QS has no direct role in PA4129-34 or *cioAB* expression is consistent with the lack of a LasR- or RhlR-binding site in the respective promoters (Gilbert et al., 2009, Schuster and Greenberg, 2007, Schuster et al., 2003, Wurtzel et al., 2012).

We do not yet know how cyanide is sensed and which regulatory pathways may be involved. In any case, the induction of resistance genes by cyanide seems ecologically prudent, as it confers protection to cyanide solely when necessary. For example, resistance will be beneficial for cells at low density that are exposed to cyanide produced by competing microbes. On the other hand, resistance will be unnecessary in high-density environments that do not result in high cyanide production rates or accumulation of endogenous cyanide, such as high aeration or high mass transfer.

Our conclusion is at odds with the initial study by Wang et al*.* that proposed the cyanide policing model (Wang et al., 2015). We briefly outline the main discrepancies. Wang et al. reportedly used high-aeration growth conditions, which, as we demonstrated here, do not produce detectable levels of cyanide (Figure 1B). They conducted long-term evolution experiments with cyanide-deficient *rhlR* or *hcnC* mutants that appear to reduce the tolerance threshold for *lasR* cheaters. Long-term culturing in general is problematic, as we have pointed out above, as additional undefined mutants evolve. The *rhlR* mutant itself is pleiotropic, because RhlR controls not only cyanide production but also a number of other genes (Schuster et al., 2003). Together with LasR, RhlR induces the expression of extracellular proteases, such as LasB elastase, that permit growth on protein medium (Mitri and Foster, 2016, Schuster et al., 2003). RhlR-deficient cooperators therefore contribute a lower level of public goods to the community, presumably decreasing the cheater threshold, which leads to population collapse. RhlR mutants also do not produce the redox-active metabolite pyocyanin, which has been shown to impair QS-deficient cheaters (Castaneda-Tamez et al., 2018). Interpretation of results obtained with their *hcnC* mutant is difficult, as defined co-cultures lacked a wild-type control. The *hcnC* mutant also behaved differently than our *hcnB* mutant. It grew substantially faster than the wild-type in protein medium under high-aeration conditions where the burden from cyanide production and resistance should be low. We did not observe such a difference in growth rates, and the underlying reason for this discrepancy is not clear.

Fundamental questions remain regarding the evolution of policing. In social insects, kin conflict appears to have driven the evolution of worker policing in most cases (Ratnieks et al., 2006). Hence, policing likely evolved to stabilize cooperation. The same could probably not be said about the evolution of cyanide production in *P. aeruginosa*, even if it were involved in poisoning *lasR*-mutant cheaters. Cyanide is a general cellular poison that inhibits respiration by binding to the enzyme cytochrome *c* oxidase, allowing cyanide-producing bacteria to harm a range of different competing microbes or eukaryotic hosts (Gallagher and Manoil, 2001, Hibbing et al., 2010). Thus, it is likely that cyanide production in bacteria evolved to increase interspecific competitive fitness or virulence.

Our work suggests that a detailed understanding of the complexity of gene regulation is essential for predicting the evolutionary stability of cooperative behavior. The potential of gene regulation architecture in stabilizing cooperation has been recognized (Mellbye and Schuster, 2014, Schuster et al., 2017, Wechsler et al., 2019, Xavier et al., 2011). It often involves the pleiotropic control of cooperative (public) and non-cooperative (private) traits by a single regulator, such that the potential benefit from cheating is negated by a cost associated with the loss of the private trait (Foster et al., 2004, Schuster et al., 2017). Examples of private traits co-regulated by QS in *P. aeruginosa* are substrate utilization and resistance to stress (Dandekar et al., 2012, Garcia-Contreras et al., 2015). However, although pleiotropy can help maintain cooperative behavior in the short-term under specific growth conditions, it cannot ultimately explain cooperation over evolutionary time scales. As we and others have posited, when a genetic architecture is allowed to evolve, mutations can break the pleiotropic linkage between public and private goods, such that cheaters again can invade cooperators (Dos Santos et al., 2018, Schuster et al., 2017). The present study illustrates precisely what happens if public and private goods are not linked and are instead controlled by separate regulatory pathways: Pleiotropic cheater control does not work. Cyanide resistance as the private trait is primarily controlled by cyanide, and is uncoupled from the direct control of cyanide and other public goods by QS.

### Limitations of the Study

Our experimental design utilizes a closed batch culture system to achieve low-aeration conditions, as described in previous studies (Frangipani et al., 2014, Zlosnik et al., 2006). In this system, growing cultures generate an oxygen-limited environment through consumption of the available oxygen, and endogenous or exogenous cyanide (mostly present as HCN gas at 37°C, neutral pH, and a pKa of 9.2) is trapped inside the culture tube. A limitation of this setup is that sampling during growth reintroduces oxygen and causes cyanide loss. Consequently, sampling was largely restricted to endpoints, and optical densities were measured in tubes non-invasively. To conduct multiple measurements throughout growth in a controlled oxygen-limited environment, a more elaborate bioreactor system, potentially combined with a cyanide-ion-selective electrode, would be required.

## Methods

All methods can be found in the accompanying Transparent Methods supplemental file.

## Supplemental Information

Document S1. Transparent Methods and Tables S1–S4 and S6

Table S5. Tukey's Post-hoc Test, Related to Figure 2
