# Supplementary material for: Bacterial Cheaters Evade Punishment by Cyanide
Source: iScience. 2019 Jul 16;19:101–9. doi: 10.1016/j.isci.2019.07.015 (PMC6664145; doi:10.1016/j.isci.2019.07.015)
Supplement: Document S1. Transparent Methods and Tables S1–S4 and S6 [file mmc1.pdf]

ISCI, Volume *19*

Supplemental Information

Bacterial Cheaters Evade Punishment by Cyanide

Parker Smith, Jamison Cozart, Bryan K. Lynn, Erin Alberts, Emanuela Frangipani, and Martin Schuster

Supplemental tables

Table S1. Two-way ANOVA related to Figs. 1A, 1C, 2, and 3

**Source of variation**	**% of total variation**	***p*-value[a]**
Fig. 1A (effects of aeration and strains)		
Interaction	6.552	0.1999
Aeration	66.53	**0.0021**
Strain	0.06776	0.8905
Fig. 1C (effects of strains and time)		
Interaction	21.89	**< 0.0001**
Time	64.81	**< 0.0001**
Strain	11.5	**< 0.0001**
Fig. 2 (effects of strains and time)		
Interaction	9.43	**< 0.0001**
Time	74.19	**< 0.0001**
Strain	14.06	**< 0.0001**
Fig. 3: PA4130 – lacZ (effects of KCN and strains)		
Interaction	38.11	**< 0.0001**
KCN	39.48	**< 0.0001**
Strain	19.8	**< 0.0001**
Fig. 3: PA4133 – lacZ (effects of KCN and strains)		
Interaction	17.27	**< 0.0001**
KCN	68.46	**< 0.0001**
Strain	9.67	**0.0011**
Fig. 3: cioA – lacZ (effects of KCN and strains)		
Interaction	2.786	0.4599
KCN	5.351	0.0996
Strain	71.71	**0.0001**
Fig. 3: hcnA – lacZ (effects of KCN and strains)		
Interaction	3.704	**0.0012**
KCN	0.2138	0.2522
Strain	94.31	**< 0.0001**

[a] *p*-values < 0.05 in bold

Table S2. One-sample Student's t-test related to Fig. 1A[a]

**Comparison**	***p*-value[b]**
WT:lasR/High aeration	**0.0091**
hcnB:lasR/High aeration	**0.0033**
WT:lasR/Low aeration	**0.0101**
hcnB:lasR/Low aeration	**0.0027**

[a] two-tailed, $n = 3$, *theoretical mean* = 1

[b] *p*-values < 0.05 in bold

Table S3. Two-sample Student's t-test related to Fig. 1B[a]

**Comparison**	***p*-value[b]**
Low aeration vs. High aeration	**0.016**

[a] two-tailed, unpaired, equal variance, $t = 4.011$, *d.f.* = 4

[b] *p*-values < 0.05 in bold

Table S4. Tukey's post-hoc test related to Fig. 1A and 1C

Comparisons	Adjusted *p*-value[a]
Fig. 1A (d.f. = 8)	
High aeration:WT:lasR vs. High aeration:hcnB:lasR	0.8116
High aeration:WT:lasR vs. Low aeration:WT:lasR	**0.0139**
High aeration:WT:lasR vs. Low aeration:hcnB:lasR	0.0622
High aeration:hcnB:lasR vs. Low aeration:WT:lasR	**0.0469**
High aeration:hcnB:lasR vs. Low aeration:hcnB:lasR	0.2139
Low aeration:WT:lasR vs. Low aeration:hcnB:lasR	0.7059
Fig. 1C (d.f. = 24)	
Time = 0h	
WT vs. hcnB	0.9895
WT vs. lasR	0.9988
hcnB vs. lasR	0.9814
Time = 6h	
WT vs. hcnB	> 0.9999
WT vs. lasR	0.9599
hcnB vs. lasR	0.9631
Time = 12h	
WT vs. hcnB	0.7556
WT vs. lasR	**0.0035**
hcnB vs. lasR	**0.0193**
Time = 24h	
WT vs. hcnB	0.2831
WT vs. lasR	**< 0.0001**
hcnB vs. lasR	**< 0.0001**

[a] *p*-values < 0.05 in bold

Table S6. Tukey's post-hoc test related to Fig. 3

Comparisons	Adjusted *p*-value[a]
PA4130 – lacZ (d.f. = 12)	
WT:No KCN vs. WT:+ KCN	0.1338
WT:No KCN vs. hcnB:No KCN	**< 0.0001**
WT:No KCN vs. hcnB:+ KCN	0.1575
WT:No KCN vs. lasR:No KCN	**< 0.0001**
WT:No KCN vs. lasR:+ KCN	0.1349
WT:+ KCN vs. hcnB:No KCN	**< 0.0001**
WT:+ KCN vs. hcnB:+ KCN	> 0.9999
WT:+ KCN vs. lasR:No KCN	**< 0.0001**
WT:+ KCN vs. lasR:+ KCN	**0.0014**
hcnB:No KCN vs. hcnB:+ KCN	**< 0.0001**
hcnB:No KCN vs. lasR:No KCN	0.8265
hcnB:No KCN vs. lasR:+ KCN	**< 0.0001**
hcnB:+ KCN vs. lasR:No KCN	**< 0.0001**
hcnB:+ KCN vs. lasR:+ KCN	**0.0017**
lasR:No KCN vs. lasR:+ KCN	**< 0.0001**
PA4133 – lacZ (d.f. = 12)	
WT:No KCN vs. WT:+ KCN	0.211
WT:No KCN vs. hcnB:No KCN	**0.0002**
WT:No KCN vs. hcnB:+ KCN	0.3039

WT:No KCN vs. lasR:No KCN	**0.0002**
WT:No KCN vs. lasR:+ KCN	**0.0036**
WT:+ KCN vs. hcnB:No KCN	**< 0.0001**
WT:+ KCN vs. hcnB:+ KCN	0.9998
WT:+ KCN vs. lasR:No KCN	**< 0.0001**
WT:+ KCN vs. lasR:+ KCN	0.2041
hcnB:No KCN vs. hcnB:+ KCN	**< 0.0001**
hcnB:No KCN vs. lasR:No KCN	> 0.9999
hcnB:No KCN vs. lasR:+ KCN	**< 0.0001**
hcnB:+ KCN vs. lasR:No KCN	**< 0.0001**
hcnB:+ KCN vs. lasR:+ KCN	0.1378
lasR:No KCN vs. lasR:+ KCN	**< 0.0001**
cioA – lacZ (d.f. = 12)	
WT:No KCN vs. WT:+ KCN	> 0.9999
WT:No KCN vs. hcnB:No KCN	**0.0066**
WT:No KCN vs. hcnB:+ KCN	**0.0485**
WT:No KCN vs. lasR:No KCN	**0.0038**
WT:No KCN vs. lasR:+ KCN	0.083
WT:+ KCN vs. hcnB:No KCN	**0.0061**
WT:+ KCN vs. hcnB:+ KCN	**0.0444**
WT:+ KCN vs. lasR:No KCN	**0.0035**
WT:+ KCN vs. lasR:+ KCN	0.0762
hcnB:No KCN vs. hcnB:+ KCN	0.8353
hcnB:No KCN vs. lasR:No KCN	0.9992
hcnB:No KCN vs. lasR:+ KCN	0.6649
hcnB:+ KCN vs. lasR:No KCN	0.6542
hcnB:+ KCN vs. lasR:+ KCN	0.9994
lasR:No KCN vs. lasR:+ KCN	0.471
hcnA – lacZ (d.f. = 12)	
WT:No KCN vs. WT:+ KCN	**0.0069**
WT:No KCN vs. hcnB:No KCN	**0.0004**
WT:No KCN vs. hcnB:+ KCN	**0.0202**
WT:No KCN vs. lasR:No KCN	**< 0.0001**
WT:No KCN vs. lasR:+ KCN	**< 0.0001**
WT:+ KCN vs. hcnB:No KCN	0.5061
WT:+ KCN vs. hcnB:+ KCN	0.9852
WT:+ KCN vs. lasR:No KCN	**< 0.0001**
WT:+ KCN vs. lasR:+ KCN	**< 0.0001**
hcnB:No KCN vs. hcnB:+ KCN	0.2199
hcnB:No KCN vs. lasR:No KCN	**< 0.0001**
hcnB:No KCN vs. lasR:+ KCN	**< 0.0001**
hcnB:+ KCN vs. lasR:No KCN	**< 0.0001**
hcnB:+ KCN vs. lasR:+ KCN	**< 0.0001**
lasR:No KCN vs. lasR:+ KCN	> 0.9999

[a] *p*-values < 0.05 in bold

Transparent methods

Strains and media

The strains used in this study are the *P. aeruginosa* PAO1 wild-type (ATCC 15692) and derived marker-less deletion mutants. The *hcnB*, *cioAB* PA4129-34, and *lasR* mutants have been described (Frangipani et al., 2014; Wilder et al., 2011). The *lasR rhlR* double mutant was generated by allelic exchange (Hoang et al., 1998), introducing an existing *rhlR* deletion construct (Wilder et al., 2011) into the *lasR* deletion background. Unbuffered LB medium and LB agar was used for routine culturing. Lennox LB medium buffered with 50 mM MOPS (pH 7.5) was used for cyanide addition experiments, as in our previous study (Frangipani et al., 2014). An M9 minimal medium containing 1% (w/v) casein as the sole carbon and energy source was used for "cooperative" culture conditions that require QS-controlled proteolysis. The medium is identical to that used in previous studies (Asfahl et al., 2015; Dandekar et al., 2012; Sandoz et al., 2007; Wang et al., 2015; Wilder et al., 2011). A 100 mM potassium cyanide (KCN) stock was prepared in 10 mM phosphate buffer, pH 9.0. Where appropriate, the antibiotics trimethoprim and tetracycline were used at concentrations of 200 µg and 100 µg, respectively.

Growth experiments in casein medium

Single and co-culturing experiments were conducted in 4 mL of M9 casein medium. High-aeration experiments were performed in 20 mL glass culture tubes, capped with standard plastic lids that permit air flow, and shaken at 250 rpm. Low-aeration experiments were performed in 20 mL screw-cap glass tubes, tightly capped to avoid cyanide loss, and shaken slowly at 150 rpm. Experimental cultures were inoculated from 18-h LB pre-cultures to an OD_{600} of 0.01. For co-cultures, the *hcnB* mutant and its wild-type parent were each mixed with the *lasR* mutant at an initial frequency of 99:1. The *lasR* mutant was tagged with a trimethoprim resistance gene cassette, inserted in single-copy at a neutral chromosomal site (Wilder et al., 2011). For successive sub-culturing, aliquots from saturated co-cultures were inoculated into fresh medium at 100-fold dilution. Cell densities of subpopulations (in CFU/mL) were quantified by dilution-plating on medium with and without trimethoprim. Relative fitness *w* was calculated as the ratio of Malthusian growth parameters, with $w = \ln(N1_0/N1_{24})/\ln(N2_0/N2_{24})$, where $N1_0$ and $N1_{24}$ designate the CFU/mL of the *lasR* mutant at 0 and 24 h, respectively, and $N2_0$ and $N2_{24}$ designate the CFU/mL of either the wild-type or the *hcnB* mutant at 0 and 24 h, respectively.

Growth experiments in LB medium with exogenous cyanide

Cultures were grown in 16 mL screw-cap glass tubes containing 4 ml of LB medium buffered with 50 mM MOPS pH 7.5. In this medium, QS mutants grow to high density in monoculture (Schuster et al., 2003). Tubes were tightly closed and agitated at 150 rpm. Experimental cultures were inoculated to an OD_{600} of 0.02 from log-phase LB pre-cultures (OD_{600} of 0.4). This low-density pre-culturing step was included to avoid potential differences in the cyanide-dependent expression of resistance factors between the wild-type and the *lasR* mutant at the beginning of the experiment. Cyanide was added to half of the cultures at a final concentration of 100 µM. The glass tubes were chosen such that optical density could be measured directly in-tube using a suitable spectrophotometer (Spectronic 20D+, Thermo Fisher). This approach avoided repeated opening of tubes for sampling, increasing chemical safety and avoiding the outgassing of cyanide.

Cyanide assay

The concentration of hydrogen cyanide in co-cultures was measured using the Spectroquant cyanide cell test (EMD Millipore). The detection limit of this colorimetric assay is 0.4 µM. To minimize the outgassing of cyanide, samples were processed immediately. Culture aliquots were centrifuged, and cell-free supernatants were processed according to the manufacturer's instructions. An optional boiling step was omitted as it did not result in an increase in the amount of liberated cyanide. To eliminate non-specific signal background in this assay, an *hcnB* mutant culture was used as blank. The *hcnB* deletion mutant does not produce any cyanide (Frangipani et al., 2014).

Gene expression measurements

To generate strains for gene expression measurements, plasmids carrying promoter-*lacZ* translational fusions were introduced into the wild-type, the *lasR* mutant, and the *hcnB* mutant by chemical transformation (Chuanchuen et al., 2002). Plasmids pME7554 (*cioA*'-'*lacZ*) and pME9317 (PA4130'-

‘*lacZ*) have been described elsewhere (Frangipani et al., 2014; Frangipani et al., 2008). A translational PA4133’-‘*lacZ* fusion was constructed by inserting an 840-bp *Eco*RI-*Bam*HI fragment carrying the proximal part of PA4133 into the same restriction sites of pME6014 (Schnider-Keel et al., 2000). This fragment was generated by PCR amplification of PAO1 genomic DNA using forward primer 5’-CGCGAATTCACGTCTACGCCGAGCTGT-3’ and reverse primer 5’-GGCGGATCCTCATCTGTACAGTCCCGAAA-3’. A translational *hcnA’*-‘*lacZ* fusion was constructed similarly by fusing a 441-bp *Eco*RI-*Hind*III fragment carrying the proximal part of *hcnA* amplified from PAO1 genomic DNA with forward primer 5’-TATGAATTCCGCACTGAGTCGGACATGA-3’ and reverse primer 5’-TATAAGCTTGAAGGTGCATTGCCCTTTCA-3’ to ‘*lacZ* in pME6013 (Schnider-Keel et al., 2000). Background expression levels from the promoter-less plasmids are essentially zero, because *lacZ* lacks the entire translation initiation region.

Cultures were grown in buffered LB medium under low-aeration conditions as described above. To accommodate the large number of samples, we chose a 96-well culturing format in deep-well blocks. Each 2 mL well contained 500 µL of medium, resulting in the same air-to-media ratio as in the larger LB glass cultures. The block was sealed with a fitted plastic cover. Experimental cultures were inoculated to an OD_{600} of 0.001 from stationary-phase LB cultures. After 3 h of growth, either potassium cyanide (25 µM final concentration) or an equal volume of buffer were added to the cultures. Cultures were incubated for another 9 h and harvested in early stationary phase. β-galactosidase levels were measured in a multi-function plate reader (Tecan M200) using the Galacton-Plus luminescence assay (Thermo Fisher). Relative gene expression activity was determined by dividing luminescence readings by OD_{600}.

Statistical analysis

Graphing and statistical analysis was performed in GraphPad Prism (GraphPad Software). To determine significant differences between biological replicates (significance level $\alpha = 0.05$), the following tests were employed: One-sample Student’s t-test (two-tailed) in Fig. 1A, two-sample Student’s t-test (two-tailed, unpaired, equal variance) in Fig. 1B, ordinary two-way ANOVA with Tukey’s post-hoc test in Figs. 1A and 3, and two-way repeated measure ANOVA with Tukey’s post-hoc test in Figs. 1C and 2. The Tukey test accounts for multiple comparisons and computes a multiplicity-adjusted *p*-value for each comparison. The complete statistical data are available in Tables S1-S6.

Supplemental references

Hoang, T.T., Karkhoff-Schweizer, R.R., Kutchma, A.J., and Schweizer, H.P. (1998). A broad-host-range Flp-FRT recombination system for site-specific excision of chromosomally-located DNA sequences: application for isolation of unmarked *Pseudomonas aeruginosa* mutants. Gene *28*, 77-86.

Chuanchuen, R., Narasaki, T., and Schweitzer, H.P. (2002). Benchtop and microcentrifuge preparation of *Pseudomonas aeruginosa* competent cells. Biotechniques *33*, 760-763.

Frangipani, E., Slaveykova, V.I., Reimmann, C., and Haas, D. (2008). Adaptation of aerobically growing *Pseudomonas aeruginosa* to copper starvation. J Bacteriol *190*, 6706-6717.

Schnider-Keel, U., Seematter, A., Maurhofer, M., Blumer, C., Duffy, B., Gigot-Bonnefoy, C., Reimmann, C., Notz, R., Defago, G., Haas, D.*, et al.* (2000). Autoinduction of 2,4-diacetylphloroglucinol biosynthesis in the biocontrol agent *Pseudomonas fluorescens* CHA0 and repression by the bacterial metabolites salicylate and pyoluteorin. J Bacteriol *182*, 1215-1225.
